# Predominance of spliceosomal complex formation over polyadenylation site selection in TDP-43 autoregulation

**DOI:** 10.1093/nar/gkt1343

**Published:** 2013-12-24

**Authors:** Sara Bembich, Jeremias S. Herzog, Laura De Conti, Cristiana Stuani, S. Eréndira Avendaño-Vázquez, Emanuele Buratti, Marco Baralle, Francisco E. Baralle

**Affiliations:** International Centre for Genetic Engineering and Biotechnology (ICGEB), 34149 Trieste, Italy

## Abstract

TDP-43 is a nuclear protein involved in many aspects of RNA metabolism. To ensure cellular viability, its expression levels within cells must be tightly regulated. We have previously demonstrated that TDP-43 autoregulation occurs through the activation of a normally silent intron in its 3′-UTR sequence that results in the use of alternative polyadenylation sites. In this work, we analyse which is the dominant event in autoregulation: the recognition of the splice sites of 3′-UTR intron 7 or the intrinsic quality of the alternative polyadenylation sites. A panel of minigene constructs was tested for autoregulation functionality, protein production and subcellular messenger RNA localization. Our data clearly indicate that constitutive spliceosome complex formation across intron 7 does not lead to high protein production but, on the contrary, to lower TDP-43 messenger RNA and protein levels. This is due to altered nucleocytoplasmic distribution of the RNA that is mostly retained in the nucleus and degraded. This study provides a novel in-depth characterization of how RNA binding proteins can autoregulate their own levels within cells, an essential regulatory process in maintaining cellular viability.

## INTRODUCTION

Cell viability relies on the correct protein concentration levels within the various cellular compartments (1) and prevents the development of disease, especially at the neuronal level (2). There are several pathways used by the cell to achieve this, with protein expression regulation at the messenger RNA (mRNA) level being one of the most common due to its ability to act in an efficient and rapid manner. This type of regulation is often seen in genes encoding for RNA binding proteins due to the fact that many of these are able to bind their own RNA. Such an arrangement, in fact, allows cells to set up effective negative feedback mechanism that will raise protein production rapidly when cellular levels drop below a critical threshold and inhibit protein production when cellular concentrations become too high. Several pathways where RNA binding proteins regulate their own expression through direct binding to their transcript have been described. These include proteins such as HuR (3), PTB (hnRNP I) (4), hnRNP L (5), hnRNP A/B (6), TIA-1/TIAR (7), SRSF3 (SRp20) (8), SRSF2 (SC-35) (9) and Tra2 (10). For recent reviews on the subject, the reader is referred to Buratti and Baralle (11) and to Yap and Makeyev (12). In the majority of these cases, the autoregulatory processes for these proteins are based on the selective triggering of a specific RNA degradation mechanism called nonsense-mediated decay (NMD) (13). Exceptions to this rule are represented by HuR (3) and possibly Tra2 (10) proteins where polyadenylation and translational mechanisms may be prevalent. Another notable exception to this NMD rule is represented by the mechanism described to occur for the nuclear factor TDP-43 (14,15).

TDP-43 was initially identified as a transcriptional regulator (16) and subsequently as a regulator of Cystic fibrosis transmembrane conductance regulator (CFTR) exon 9 splicing (17). The importance of TDP-43 in the neurodegeneration field was established in 2006 when it was described as the major protein component of the intracellular inclusions occurring in the neuronal tissues of patients affected by amyotrophic lateral sclerosis and frontotemporal dementia (18,19). In the patient’s affected neurons, TDP-43 is abnormally mislocalized in the cytoplasm, ubiquitinated, hyperphosphorylated and cleaved to generate C-terminal fragments (20). Currently, one hypothesis is that such mislocalization plays a pivotal role in neurodegeneration through the loss of proper TDP-43 functions in the nucleus, although gain-of-function mechanisms may be active as well (21–26).

**Figure 1.**
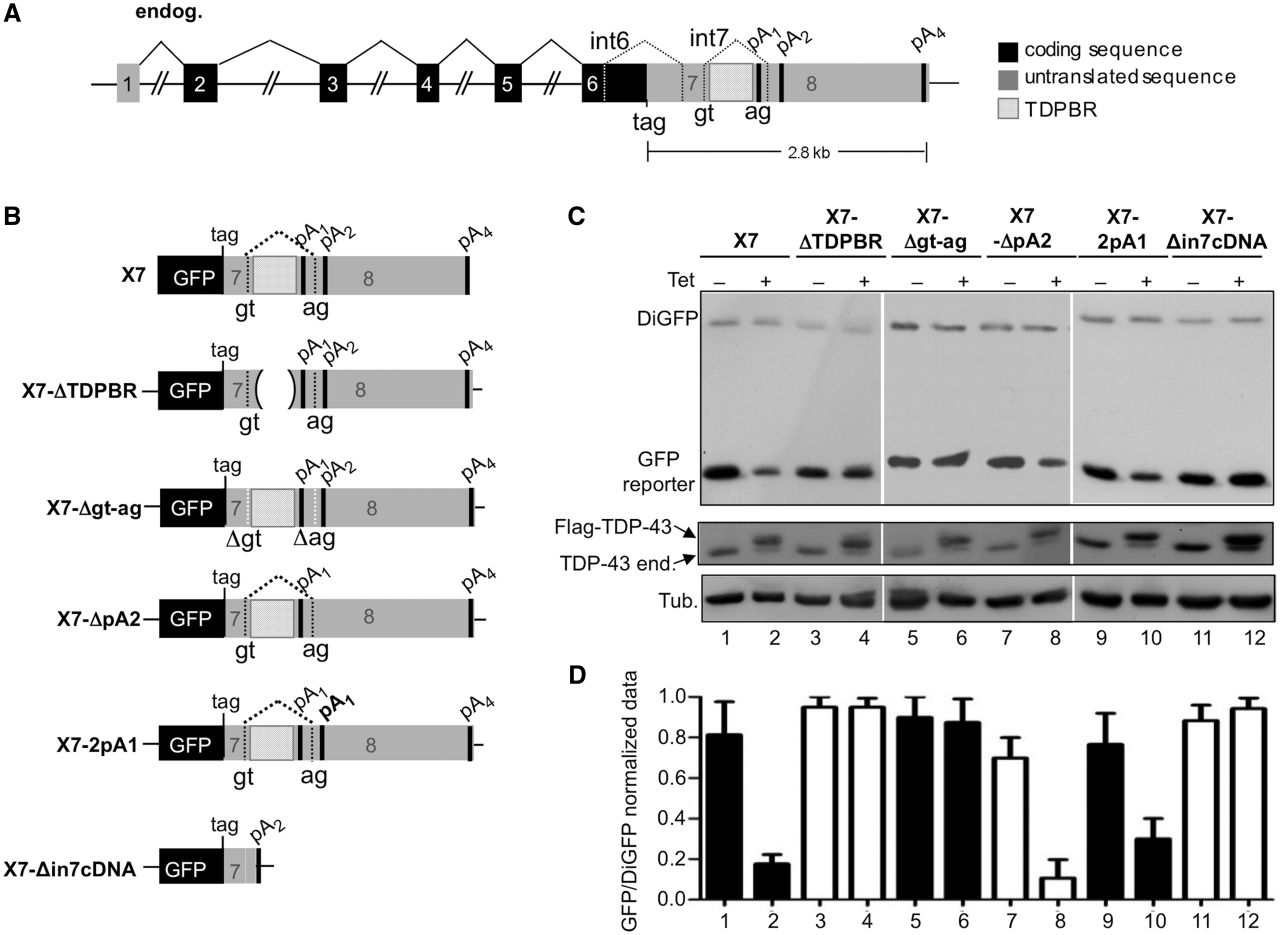
Cis acting elements and importance of PAS sequences in TDP-43 autoregulation**.** (**A**) shows a schematic diagram of TDP-43 illustrating locations of stop codon (tag), PASs (pA_1–4_), TDPBR region and splicing events (in coding sequences by filled lines; in the 3′-UTR region by dotted lines). Coding regions (black boxes), untranslated sequences (grey boxes) and introns (connecting black lines) are indicated. A schematic representation of each reporter used in this experiment is shown in (**B**). (**C**) shows the ability to autoregulate of these various TDP-43 3′-UTR constructs fused to the GFP protein and transfected in HEK-293 cells stably expressing a TDP-43 transgene following tetracycline induction (+Tet lanes). Western blots of GFP reporter protein, in normal (−Tet) and TDP-43 overexpression conditions (+Tet) are shown in the centre. With regards to the technical aspects of these experiments, it should be noted that in all these cases autoregulation efficiency of the GFP reporters was normalized by cotransfection with a plasmid expressing DiGFP (C; DiGFP, upper panel) and further confirmed by western blot against tubulin (C, lower panels). Normalization using tubulin levels, in fact, yielded similar results to those obtained using diGFP (data not shown). In parallel, correct overexpression of the TDP-43 transgene (Flag-TDP-43) and downregulation of the endogenous TDP-43 (TDP-43 end) was also assessed (C, lower panels). Finally, (**D**) shows the quantification of three independent experiments to quantify GFP protein expression levels in −Tet and +Tet conditions normalized according to DiGFP expression. Mean values are reported on the bar chart, and error bars indicate standarddeviation from at least three independent experiments.

The autoregulatory process of TDP-43 is totally dependent on a region called TDP-43 binding region (TDPBR) that contains several Cross-Linking and Immunoprecipitation (CLIP) sequences that act as targets for TDP-43 binding (27). This region is localized in TDP-43 3′-UTR and spans a normally silent intron 7 that contains the pA_1_ site (Figure 1A). In steady state conditions, pA_1_ is the major polyadenylation site (PAS) used by the TDP-43 mRNA. The pA_4_ site is also used, however, to a much lower extent.

We have previously shown that a 3-fold increase in TDP-43 nuclear levels results in an increased occupancy of the TDPBR that in turn causes a rise in polymerase II density in this sequence (15). Such a pausing effect induces an increased processing of intron 7, an effect that has been observed to occur in many experimental systems (28). Removal of intron 7 and hence of pA_1_ enforces pA_2_ and pA_4_ selection, and mRNAs using these PAS were shown to be partially retained in the nucleus (15). As a result of all these events, in the presence of high TDP-43 concentrations, a reduction of TDP-43 cellular mRNA levels occurs quickly. The relative importance in this process of TDP-43 binding to the TDPBR, spliceosome assembly, splicing of intron 7 and pA_1_-pA_2_-pA_4_ polyadenylation is still unclear. In this work, our results show that intron 7 processing and/or spliceosome assembly induced by TDP-43 is the key event in the reduction of the amount of TDP-43 mRNA rather than the intrinsic quality of the various PASs.

## MATERIALS AND METHODS

### Cell lines

The HEK-293 TDP-43 wild-type inducible cell line has been previously described (14). Cells were cotransfected using the calcium phosphate method with 3 µg of each Green Fluorescent Protein (GFP) construct variants and 0.5 µg DiGFP, generously donated by Dr Christian Beetz. It should also be noted that the reliability of our experimental conditions in the transfection assays to correctly measure GFP expression levels was determined by making a titration curve that show a proportional increase in the signal using increasing concentrations of cellular extracts from our reference X7 construct (Supplementary Figure S1). 

### Plasmid construction, 3′ RACE and reverse transcriptase-polymerase chain reaction analysis

The X7 wild-type and ΔTDPBR recombinant constructs have been previously described (15). The other X7 construct variants were made by polymerase chain reaction (PCR) amplification using quick change (Stratagene) according to manufacture’s instructions (primer sequences are available upon request). For 3′RACE (Rapid amplification of cDNA ends) analysis total RNA was reverse-transcribed using an oligonucleotide (dT)_20 _V anchor (5′-ctagtctagatctgaatatattcgttttttttttttttttttttv-3′) and amplified with the anchor (5′-cgaatatattcagatctagactag-3′) and GFPfw (5′-tctcggcatggacgagctgtacaag-3′). Cycloheximide (CHX) treatment was performed by adding 50 µg/ml of this antibiotic for 3 h before mRNA extraction.

### Immunoblotting

Cells extracts were prepared in 15 mM HEPES, pH 7.5, 0.25 M NaCl, 0.5% NP-40, 10% glycerol, 1× protease inhibitor (Roche 1873580), 25 mM NaF, 10 mM β-glycerolphosphate, 0.2 mM Na_3_VO_4_ and 1 mM phenylmethylsulphonyl fluoride. Proteins were separated by sodium dodecyl sulphate–polyacrylamide gel electrophoresis and transferred to nitrocellulose (0.45 mM, Amersham Biosciences), and protein detection was carried out with standard western blotting techniques using an antibody to detect GFP (Santa Cruz Biotechnology sc-8334). 

### Northern blotting

For northern blotting, RNA was isolated with EuroGoldtriFast (Euroclone) following manufacturer’s instructions. RNA samples were loaded on 1.2% formaldehyde agarose gels, transferred to Hybond-N^+^ nylon membranes (Amersham Biosciences) and probed with internally ^32^P-labelled sequences following prehybridization in ULTRAhyb® Ultrasensitive Hybridization Buffer (Ambion). Prehybridization and hybridization were carried out at 65°C. The probes were generated by PCR using primers GFP813FW (5′-cggcgtgcagtgcttcagccgctac-3′) and GFPend RV (5′-cttgtacagctcgtccatgccgagag-3′) and ^32^P-labelled with Rediprime II DNA Labeling System (GE Healthcare). Visualization of transcripts was carried out with a Cyclone Plus Storage Phosphor Scanner that included OptiQuant Software (Perkin Elmer).

### RNA *in situ* hybridization

HEK-293 FlpIn cells were fixed for 30 min at room temperature in 2% Paraformaldehyde (PFA) 24 h post-transfection. After three washes in phosphate-buffered saline cells were permeabilized by treatment with 0.1% triton in phosphate-buffered saline for 5 min and hybridized overnight at 37°C in 20 µl of a mixture containing 10% dextran sulphate, 10 µg yeast tRNA, 1× SSC, 20% formamide and 20 ng of digoxigenin-11-dUTP (Cat. No. 11573152910, Roche Applied Science) labelled PCR product probe against GFP coding sequence. Cells were then washed three times in 2× SSC and once in 4× SSC and incubated for 1 h at room temperature with 1:200 anti-digoxigenin-rhodamine Fab fragments (Cat. No. 11207750910, Roche Applied Science) in 4× SSC. Following three washes in 4× SSC, cells were mounted and the images were captured on a Zeiss LSM 510 META confocal microscope (Carl Zeiss Microimaging, Inc.) with a 63 × NA 1.4 Plan-Apochromat oil objective. Cells nuclei were visualized using TO-PRO-3 (Invitrogen, T3605).

## RESULTS

### Evaluating the effects of PAS shuffling on TDP-43 autoregulation

Figure 1A shows a schematic representation of the *TARDBP* gene illustrating the locations of the stop codon (tag), the various PASs (pA_1–__4_) and the TDPBR region that regulates intron 7 splicing within the non-coding 3′-UTR. This region represents an essential element for the negative feedback loop that allows TDP-43 to control its own expression levels within cells (14,15). We have previously shown that a minimal transcript consisting in a portion of the TDP-43 3′-UTR region fused to the GFP reporter protein displays similar autoregulatory properties to those observed for endogenous TARDBP gene (Figure 1B, X7 construct) (14,15). In fact, production of the GFP reporter protein from this transcript following transfection in HEK-293 cells that carry an integrated tetracycline (Tet)-inducible TDP-43 complementary DNA (cDNA) is ∼4-fold decreased on overexpression of TDP-43 (+Tet conditions) as determined by western blot analysis (Figure 1C, GFP reporter, lanes 1–2) and densitometric quantification (Figure 1D). Using this minimal transcript, we have previously reported the central role played by the TDPBR sequence and by the splice sites of intron 7. This was achieved by engineering mutants where the TDPBR was deleted (Figure 1B, mutants X7-ΔTDPBR) and another construct where the splice sites were disrupted (Figure 1B, X7-Δgt-ag respectively) (15). As shown in Figure 1C, no GFP downregulation could be observed following tetracycline induction of TDP-43 expression for neither of these mutants (Figure 1C, lanes 3–6 and Figure 1D). 

A question that remained open from these experiments was the eventual role played by pA_4_ in TDP-43 autoregulation process. Therefore, to assess its eventual importance, we engineered a mutant GFP reporter (Supplementary Figure S2A, X7-inpA2) in which the entire TDP-43 3′-UTR sequence was deleted from 33 nt beyond pA_2_ till the pA_4_ and compared its protein expression levels with those of X7 both in −Tet and +Tet conditions. This experiment showed that in +Tet conditions the X7-inpA2 could efficiently autoregulate its own levels similarly to the wild-type X7 construct (Supplementary Figure S2B), even in the absence of pA_4_, indicating that this PAS does not contribute significantly to the TDP-43 autoregulatory process. Further work is required to understand the biological function of the 3′-UTR beyond pA_2_.

What remained unclear from our previous results, however, was an assessment of the relative importance of PAS switch from pA_1_ to pA_2_ compared with the actual event of intron 7 processing. To address the relative importance of pA_1_ and pA_2_ in the autoregulatory process, we engineered two mutants, one in which the pA_2_ was removed (X7-ΔpA2) and another in which pA_2_ was replaced with a second pA_1_ site (X7-2pA1) (Figure 1B). Following transfection of these mutants in HEK-293 cells (−Tet lanes) and induction of the transgene (+Tet), it was observed that both constructs displayed efficient autoregulation (Figure 1C, lanes 7–8 and 9–10, respectively, and 1D). These results indicate that the use of pA_2_ is not intrinsically required to achieve TDP-43 autoregulation, and that it is not inherently defective but capable of sustaining efficient protein translation. To further corroborate this, we engineered a mutant X7 construct, X7-Δin7cDNA (Figure 1B), in which intron 7 was removed artificially and the pA_2_ sequence was kept as the only available PAS (artificially mimicking the transcript that would naturally originate following intron 7 removal by the spliceosome). Following transfection in HEK-293 cells of this mutant, it was observed that X7-Δin7cDNA produced TDP-43 at an analogous level than X7 in −Tet conditions (Figure 1C, lane 11 and Figure 1D). Moreover, in contrast X7, it was no longer capable of autoregulation following transgene induction (Figure 1C and D).

### The 3′RACE analysis of the PAS-modified constructs

**Figure 2.**
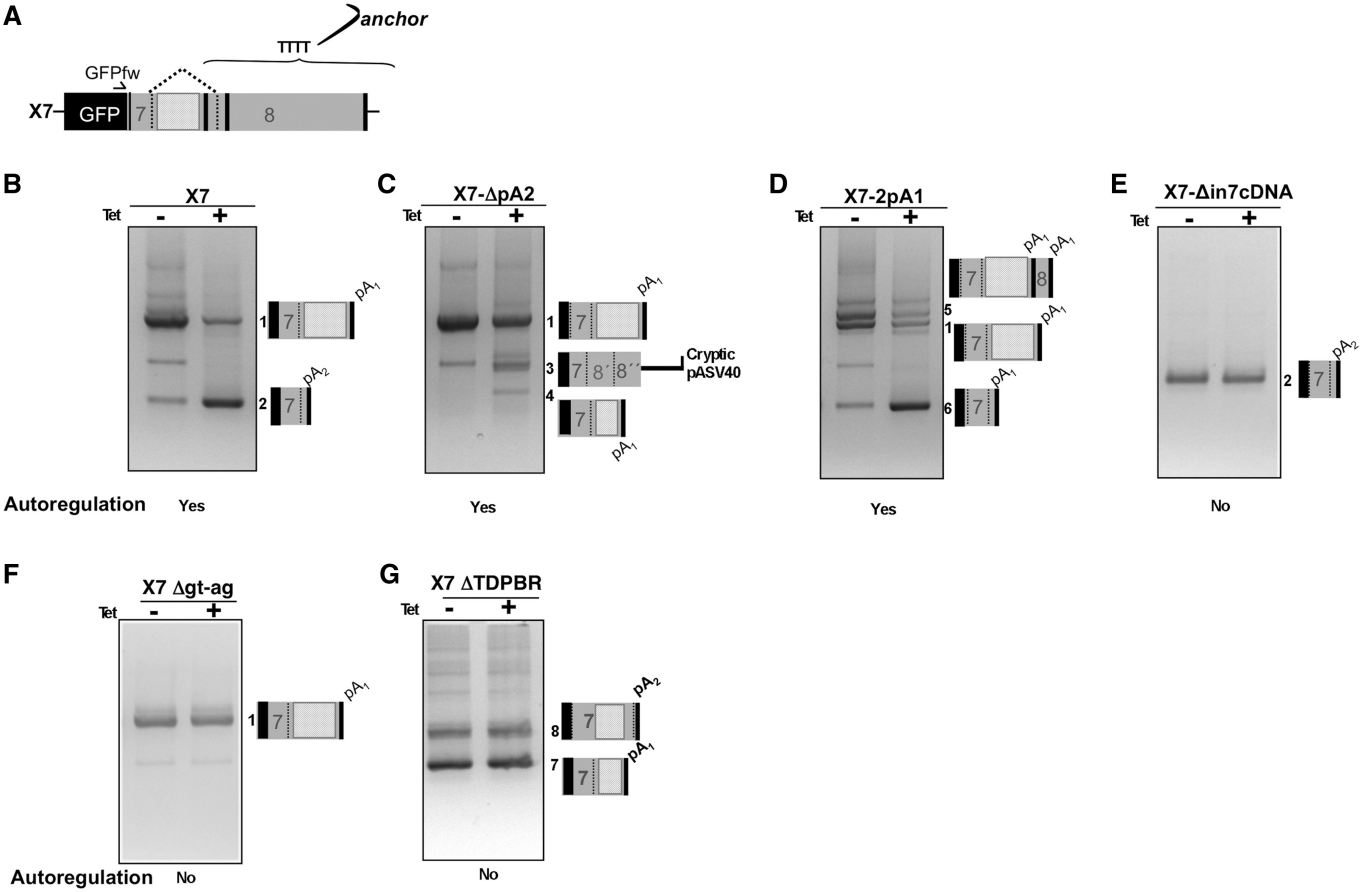
3′ RACE analyses**.** (**A**) shows a schematic diagram of the X7 reporter construct with an indication of the primers used for 3′ RACE analysis (GFPfw and anchor primers). (**B–G**) show the results of the 3′ RACE analyses from the various hybrid constructs under normal (−Tet) and following TDP-43 transgene overexpression (+Tet). The amplified bands were all subjected to sequencing and the schematic diagrams on the right show which PAS become activated following transgene induction.

To gain better insight regarding PAS usage in these constructs, 3′ RACE analysis with primers specific for the GFP reporter sequence (GFPfw) and a polyT anchor as reverse primer was performed (Figure 2A). As previously reported for the X7 plasmid (15), in normal conditions a dominant transcript was obtained (band 1, −Tet lane) that contained the TDP-43 3′-UTR sequence ending at pA_1_ (Figure 2B). On induction of the transgene, the X7 band 1 diminished and a new product appeared (band 2) that was observed to derive from intron 7 removal and the use of pA_2_ (Figure 2B, +Tet lane).

A decrease of band 1 was also obtained for the other two constructs, X7-ΔpA2 and X7-2pA1 that displayed autoregulation at the protein level following transgene induction. In fact, Figure 2C–D shows that in both cases the transgene induction caused a drop in the mRNA that contain the unspliced intron 7 and resulted in the appearance of novel mRNA (compare −Tet and +Tet lanes). Sequencing of these bands revealed that these spliced transcripts used alternative PAS. In the case of X7-ΔpA2, the decrease in band 1 is associated with the use of a cryptic PAS within the plasmid after unusual splicing events within the 3′-UTR (band 3, Figure 2C), and one originating from a cryptic splicing event within intron 7 (band 4, Figure 2C). X7-2pA_1_ on the other hand uses the second pA_1_ after the intron 7 3′ss that is uniquely present in this mutant (bands five and six, Figure 2D). In fact, the X7-2pA_1_ construct has the option of using also the second pA_1_ (placed at the site of pA_2_) in −Tet conditions when there is no intron 7 splicing. As a substantial amount of transcript made use of this second pA_1_ sequence, it would appear that the pA_1_ is more efficient than pA_2_, with run-through transcripts that do not use the first pA_1_ using the second pA_1_ as well, possibly also detrimental to pA_4_ use (that cannot be detected in this 3′RACE analysis because it is >3 kb away, and a fragment this long does not amplify as efficiently as the shorter ones). 

The 3′ RACE of the X7-Δin7cDNA showed that the pA_2_ contained in this construct was used in all the mRNAs transcribed both under −Tet and +Tet conditions, and that no further processing of this transcript could be observed (Figure 2E). This situation is similar to that previously described in the X7-Δgt-ag mutant that does not autoregulate even if in the case the pA_1_ is used (15) (Figure 2F). In this mutant, intron 7 splicing is completely inhibited following the inactivation of its donor and acceptor splice sites. As a result, the only transcript observed for this construct uses pA_1_ in a constitutive manner, both in −Tet and +Tet conditions (band 1, Figure 2F). A similar situation is also observed for the X7-ΔTDPBR construct in which deletion of the TDPBR region leads to loss of autoregulation (Figure 2G).

These constructs allow us to conclude that autoregulation is not strictly linked to the quality of the PASs. Furthermore, it is interesting to note that autoregulation only takes place when splicing is occuring, suggesting that the key feature that allows TDP-43 autoregulation is represented by spliceosome assembly on intron 7 splice sites.

### Comparing the mRNA and protein expression levels of the X7-Δin7cDNA and X7 constructs

One of the most interesting findings of the mutants analysed in Figure 1 was the relatively equal protein production stemming from differential pA_1_ and pA_2_ usage by the X7 and X7-Δin7cDNA (compare Figure 1B, lanes 1 and 11 and the −Tet lanes in Figure 2B and E). In −Tet conditions, in fact, the X7 construct uses almost exclusively pA_1_ as opposed to X7-Δin7cDNA that uses exclusively the pA_2_ site. This allowed us to conclude that the use of pA_2_ is not the main factor responsible for the autoregulation process.

To analyse further eventual functional differences in mRNAs carrying these two different PASs exists, we then decided to compare the level of mRNA in cells without transgene induction for the constructs X7 and X7-Δin7cDNA.

**Figure 3.**
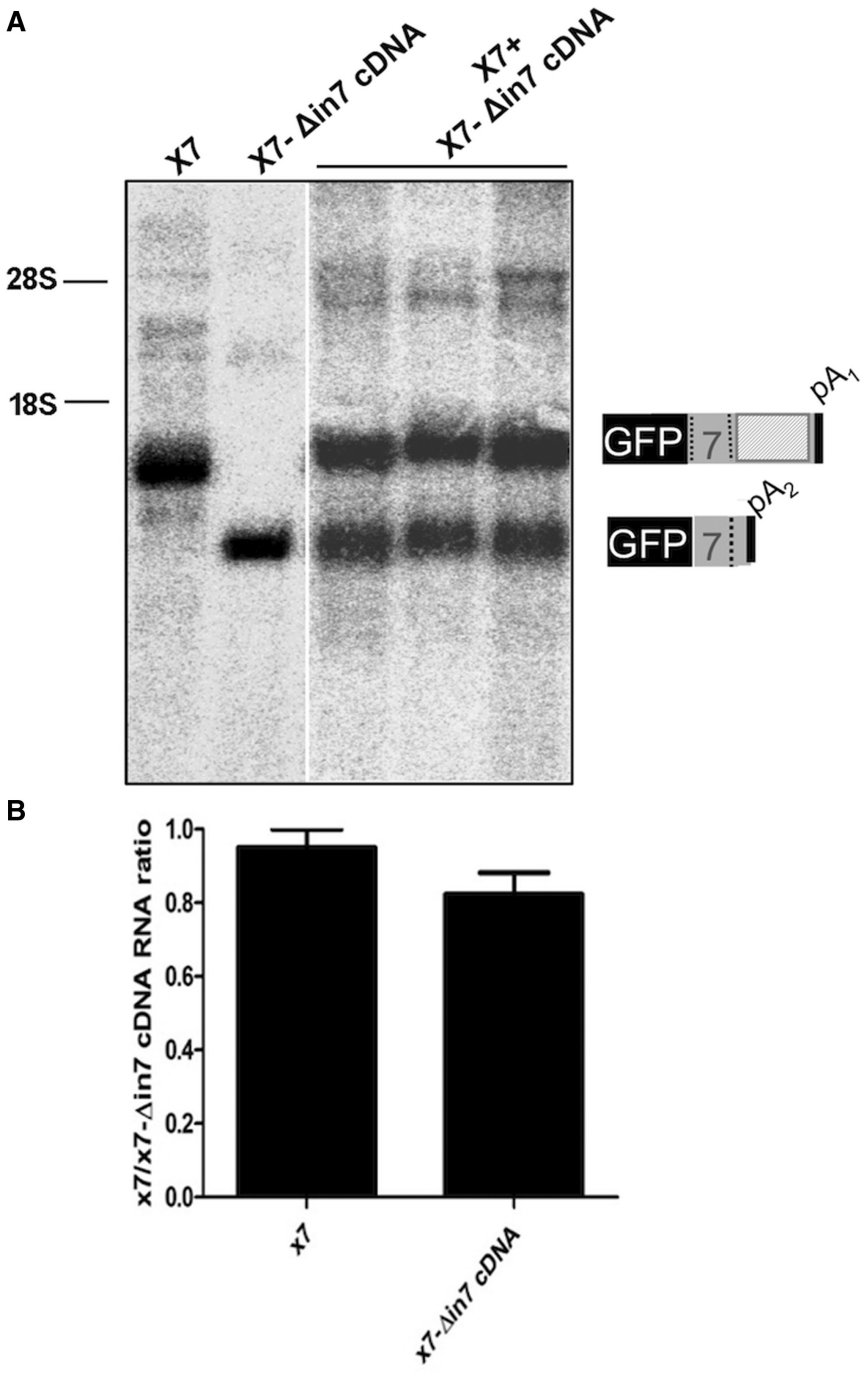
Comparison of RNA and protein levels in the X7-Δin7cDNA and X7 constructs. (**A**) shows the northern blots following transfection of the X7-Δin7cDNA and X7 constructs in HEK-293 cells under −Tet conditions. Both plasmids were transfected alone and together (in triplicate) to allow for normalization. Schematic diagrams for each band are shown on the right. (**B**) shows the quantification of the RNAs derived from X7-Δin7cDNA normalized to the intensity of the X7 transcript. Standard deviations from the three independent experiments are reported.

The comparative RNA expression levels from these two constructs were analysed by northern blot following transfection of the two constructs in HEK-293 cells either separately or together (Figure 3A). Quantification of the various band intensities detected only a slightly lower mRNA level of the X7-Δin7cDNA construct with respect to the X7 constructs (Figure 3B). However, this decrease failed to reach statistical significance consistent with protein expression levels (Figure 1, lanes 1 and 11).

Therefore, taken together these results suggest that the use of pA_1_ instead of pA_2_ does not result in any significant difference with regards to mRNA and protein expression levels, and that the two PASs are perfectly comparable in terms of protein production efficiency.

### Improving intron 7 splice site strength to induce its constitutive splicing

The central importance of intron 7 in the autoregulation process of TDP-43 was previously shown in a X7 Δgt-ag mutant, in which loss of autoregulation following expression of the transgene was achieved by simply deleting both splice sites from the wild-type X7 construct (15) and Figure 1, lanes 5 and 6.

**Figure 4.**
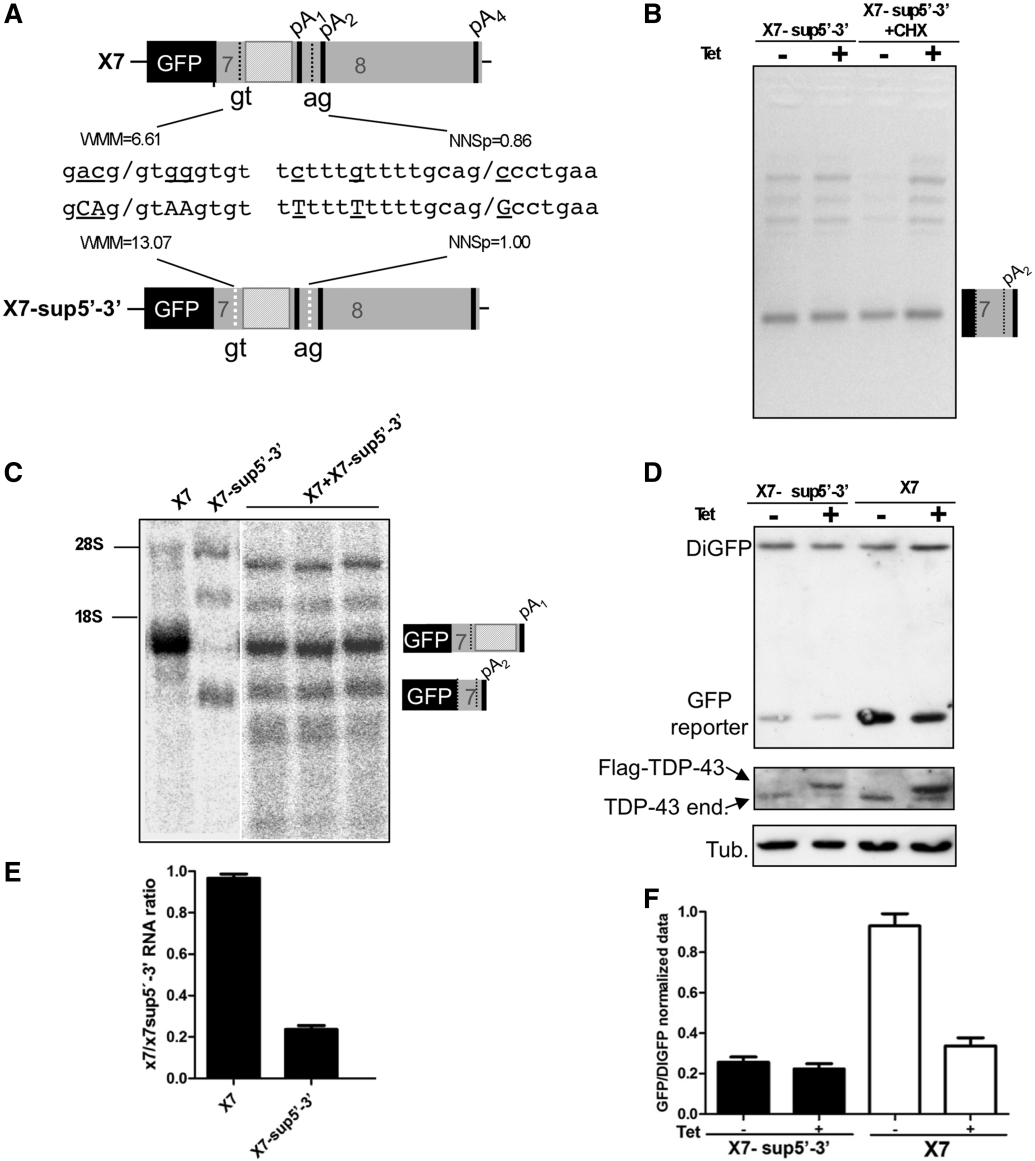
Effects on mRNA and protein level production of improving intron 7 donor and acceptor splice sites. (**A**) shows a schematic diagram of the intron 7 splice sites both in their natural setting (X7) and in the improved version (X7-sup5′-3′). (**B**) shows a 3′ RACE analysis of X7-sup5′-3′ either in normal conditions (−CHX) or following CHX treatment (+CHX) to rule out eventual effects by NMD. (**C**) shows a northern blot analysis of X7 and the X7-sup5′-3′ mutant transfected alone or together (in triplicate) to allow for quantification (**E**). With regards to GFP protein production, (**D**) shows the immunoblots of the X7-sup5′-3′ construct with respect to the X7 construct under −Tet and +Tet conditions. As in Figure 1, these constructs were cotransfected with DiGFP to allow for internal normalization. Below this figure, a western blot is also reported to show the correct overexpression of the transgene (Flag-TDP-43) and the consequent shut down of the endogenous TDP-43 (TDP-43 end) in the +Tet conditions. Finally, all western blots were also hybridized with an antibody against tubulin to act as a further normalizing control. (**F**) shows the quantification of three independent experiments of the X7-sup5′-3′ versus X7 protein expression levels (error bars indicate standarddeviation from at least three independent experiments).

To complement the X7 Δgt-ag mutant result and further extend our insight into the role of splicing of this intron in autoregulation, it was then decided to mutate both these sequences to improve the strength of the splice sites. In this respect, it should be noted that in normal conditions intron 7 splicing is not observed. This is not surprising, as its 5′ and 3′ splice sites are not of optimal strength according to Maxentscan weight matrix model (29) and NNSplice (30) (Figure 4A, upper schematic diagram). A considerable improvement was then achieved in mutant X7-sup5′-3′ (Figure 4A, lower schematic diagram). The purpose of these mutations was to make the splicing of intron 7 as constitutive as possible without the need for any external enhancer factor (like high levels of TDP-43).

As shown in the 3′RACE analysis of Figure 4B, this objective was successfully achieved and transfection in −Tet conditions immediately led to the appearance of a totally processed transcript in which pA_2_ was used as the only PAS available. As expected, this situation was unchanged following transgene induction (Figure 4B, +Tet lane). In addition, the transcripts originated from X7-sup5′-3′ are not subjected to any change following CHX treatment (Figure 4B, CHX, −Tet and +Tet lanes).

In terms of the qualitative nature of the transcript species being generated, the mRNA is the same as the one produced by the X7-Δin7cDNA transcript (compare Figure 4B with Figure 2E) with the difference that the mRNA of X7-sup5′-3′ is generated by transcription followed by splicing, whereas the mRNA of X7-Δin7cDNA only by transcription. However, quantitatively the results were strikingly different, as in northern blot analysis the amount of pA_2_-using transcript from X7-sup5′-3′ was significantly lower when compared with X7 (Figure 4C–E).

Taking in consideration the reduction in the amount of total RNA for the X7-sup5′-3′ construct (Figure 4C–E), it was then interesting to measure whether these differences could result in a reduction of GFP protein expression. In keeping with this hypothesis, the western blot result reported in Figure 4D showed that the amount of GFP protein produced by the X7-sup5′-3′ construct was much lower with respect to the X7 wild-type construct, both in −Tet and +Tet conditions. Quantification of this drop in expression confirmed that X7-sup5′-3′ GFP protein levels were ∼25% of normal X7 GFP production levels (Figure 4F), a significant reduction compared with the estimated change in mRNA levels suggesting a difference in translational ability and/or in nuclear export of the pA_2_ mRNA. 

### Altered nucleocytoplasmic distribution of the X7-sup5′-3′ transcript

**Figure 5.**
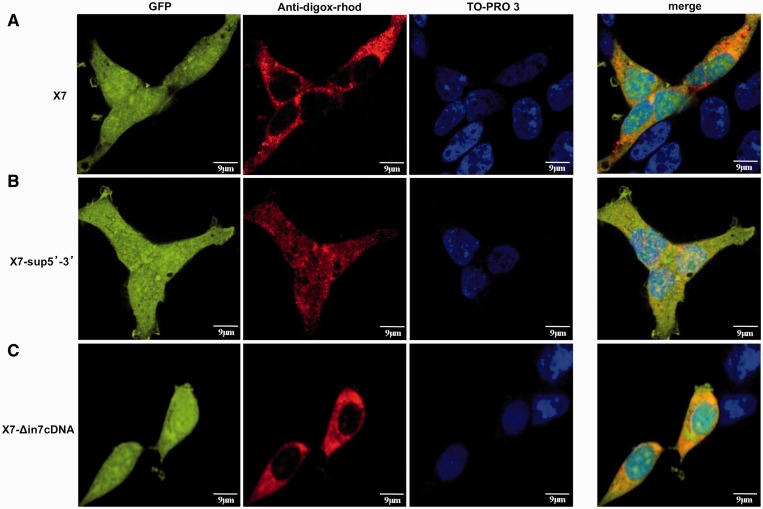
Cellular distribution of GFP different constructs RNA. (**A**) First column shows the GFP protein cellular localization of the different constructs where it can be seen that the signal is homogeneously distributed between nucleus and cytoplasm. (**B**) The second column shows the RNA cellular distribution of the constructs. Specific RNA signal is mainly cytoplasmic for the X7 and the X7-Δin7cDNA constructs, whilst it is present also in the nucleus for the X7-sup5′-3′ construct. Specific RNA detection was performed using anti-digoxigenin-rhodamin Fab fragments as described in the ‘Materials and Methods’ section. (**C**) Finally, the third column shows cells nuclei stained with TO-PRO-3. The panels on the right show the merge of these three signals. Scale bar 9 µm.

One possibility to explain the differences mentioned earlier in the text may lie on the availability of the mRNAs in the cytoplasm. RNA fluorescence *in situ* hybridization analysis of the various transcripts originating from the X7, X7-Δin7cDNA, and X7-sup5′-3′ constructs was then performed. This analysis is not suited to detect quantitative differences in expression but rather, differential distributions of RNAs in the cell body. As shown in Figure 5A by the Anti-digox-rhod signal, in red, the transcripts from X7 are predominantly localized in the cytoplasm with a consequently high efficiency of GFP production. A similar situation is also observed for the x7-Δin7cDNA construct (Figure 5C), further highlighting our observation that use of pA_1_ (X7) or pA_2_ (X7-Δin7cDNA) does not make any difference to the resulting protein production process.

On the other hand, the RNA transcribed from the X7-sup5′-3′ shows a very different nucleus/cytoplasm ratio from the one observed in both X7 and X7-Δin7cDNA notwithstanding the latter being identical to the transcript produced by X7-sup5′-3′ (Figure 5B). This data, in fact, clearly shows that instead of being mainly localized in the cytoplasm the RNA produced by X7-sup5′-3′ is uniformly distributed between the nucleus and cytoplasm. This result suggests that the 3′-UTR intron 7 recognition by the splicing machinery somehow marks the bulk of the transcript for nuclear retention and degradation, resulting in lower amounts of cytoplasmic mRNA and hence protein level.

## DISCUSSION

TDP-43 is an ubiquitously expressed protein (17,31,32), whose production is tightly autoregulated in cells (33). Even though the exact identification of the various roles played by TDP-43 has not proved to be an easy task (34), there is no doubt that correctly regulating TDP-43 concentrations within cells is essential for maintaining cell viability. It has been recently described that TDP-43 levels and intracellular localization can be transiently altered following axotomy (35), nerve crush (36), axonal ligation (37), acute ischemic stroke in rats (38) and human traumatic brain injury (39), as part of a normal physiological response to injury. Therefore, the presence of an RNA autoregulatory mechanism can help to control all these tasks quickly and then swiftly return to steady-state expression levels once the need is over. In addition, keeping a tight control on TDP-43 may also be especially important in a pathological setting, as already discussed in detail elsewhere (11,40). In this case, in fact, pathological aggregates may act as a protein ‘sink’ of TDP-43, lowering its nuclear levels and triggering through the TDP-43 self-regulation loop and ever increasing rate of TDP-43 production that might prove harmful to cells, especially neurons.

The results presented in this manuscript advance our knowledge of the mechanism of TDP-43 self-regulation in several ways. Previously, we have demonstrated that TDP-43 autoregulation involves the activation of the normally silent intron 7 in its 3′-UTR sequence, and that removal of this intron results in the use of alternative PAS (14,15). However, the importance of each event was not clear, as originally it was thought that pA_1_ and pA_2_ had intrinsic different qualities, with pA_2_ usage being detrimental to proper nuclear export and/or translation. Now, the results presented in this work clearly indicate that constitutive splicing of intron 7 does not lead to high protein production but, on the contrary, to lower mRNA and protein levels. In addition, we show here that an mRNA ending in pA_2_ that is produced only by transcription without involving spliceosome assembly does not autoregulate. Furthermore, this mRNA is exported to the cytoplasm and translated producing equivalent levels of mRNA (ending in pA_1_, Figure 3) and protein than the wild-type X7 configuration (Figure 1, compare lanes 11 and 1). Instead, the construct where the splicing of intron 7 is constitutive (X7-sup5′-3′) results in low mRNA and protein production (Figure 4B). Of course, additional modifier factors may be acting at the level of splicing and/or transcription and can also have an impact on regulating TDP-43 expression levels within cells. In fact, the splicing of introns is generally a co-operative process that involves many auxiliary factors in determining the final outcome (41–43).

These results refine substantially the previous proposal of the self-regulation determinants and mechanism. The spliceosome assembly in intron 7 either induced by TDP-43 binding to the TDPBR or by improvement of intron 7 splice sites is responsible of the downregulation of mRNA and protein production.

**Figure 6.**
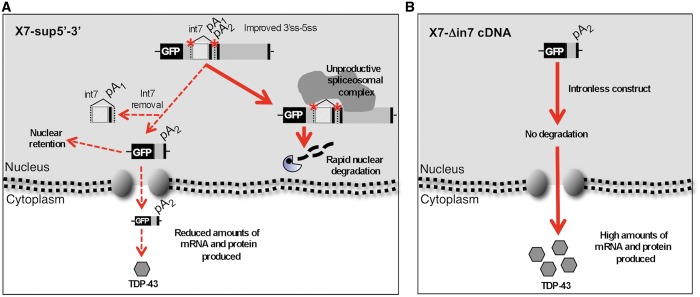
Schematic representation of the importance of intron 7 processing in autoregulation. (**A**) shows a schematic representation of the processing events of the X7-sup5′-3′ construct. In this construct, spliceosomal assembly and/or constitutive processing of intron 7 leads to degradation of the resulting transcript through a NMD-independent mechanism and inefficient export to the cytoplasm. As detected by 3′ RACE analysis, a small amount of transcripts results in pA_2_ usage that is also nuclear retained (as observed in immunocytochemistry). This results in a reduced amount of protein production. (**B**) shows the opposite situation, where intron 7 is removed artificially in the X7-Δin7cDNA construct before transfection into cells. In this case, pA_2_ usage does not cause degradation/export problems and leads to efficient TDP-43 protein production. In both 6A and B the coding regions (black boxes), untranslated sequences (grey boxes) and various PAS are indicated.

These data suggest that the autoregulation mechanism can be interpreted as described in Figure 6A. In this figure, we propose that most of the spliceosomal complex is not productive and the pre-mRNA transcript is not fully processed. As a consequence, it is retained in the nucleus and degraded. The molecules that do undergo splicing are either exported to the cytoplasm, translated and represent the source for the reduced protein production or, as we have shown before, the mature pre-mRNA bearing pA_2_ produced by the splicing process is marked somehow for substantial nucleus retention and degraded (Figure 6A). On the other hand, in the case of cDNA expression (Figure 6B) where the spliceosome is not involved, the pA_2_ mRNA is exported to the cytoplasm with reasonable efficiency and translated to protein, giving similar amount of protein compared with the pA_1_ mRNA. This latter mRNA is always produced by transcription and polyadenylation without involvement of the intron 7 splicing process when intracellular levels of TDP-43 are normal.

In conclusion, our data indicate that in the endogenous gene the key event that mediates autoregulation of TDP-43 mRNA levels in the nucleus is represented by the spliceosomal assembly process across 3′-UTR intron 7 induced by TDP-43 binding to the TDPBR.

## SUPPLEMENTARY DATA

Supplementary Data are available at NAR Online.

## FUNDING

Associazione Ricerca Italiana sulla Sclerosi Laterale Amiotrofica (AriSLA) [TARMA to F.B.] and Thierry-Latran Fondation [project REHNPALS to E.B.]. Funding for open access charge: AriSLA (Associazione Ricerca Italiana sulla Sclerosi Laterale Amiotrofica).

*Conflict of interest statement*. None declared.

## Supplementary Material

Supplementary Data
