# Supplementary material for: Predominance of spliceosomal complex formation over polyadenylation site selection in TDP-43 autoregulation
Source: Nucleic Acids Res. 2013 Dec 24;42(5):3362–71. doi: 10.1093/nar/gkt1343 (PMC3950720; doi:10.1093/nar/gkt1343)
Supplement: Supplementary Data [file supp_42_5_3362__index.html]

Predominance of spliceosomal complex formation over polyadenylation site selection in TDP-43 autoregulation — Supplementary Data 

# Predominance of spliceosomal complex formation over polyadenylation site selection in TDP-43 autoregulation

## Supplementary Data

files

**Files in this Data Supplement:**

- Supplementary Data - doc file
