# Supplementary material for: Predominance of spliceosomal complex formation over polyadenylation site selection in TDP-43 autoregulation
Source: Nucleic Acids Res. 2013 Dec 24;42(5):3362–71. doi: 10.1093/nar/gkt1343 (PMC3950720; doi:10.1093/nar/gkt1343)
Supplement: Supplementary Data [file supp_gkt1343_nar-03299-a-2013-File008.doc]

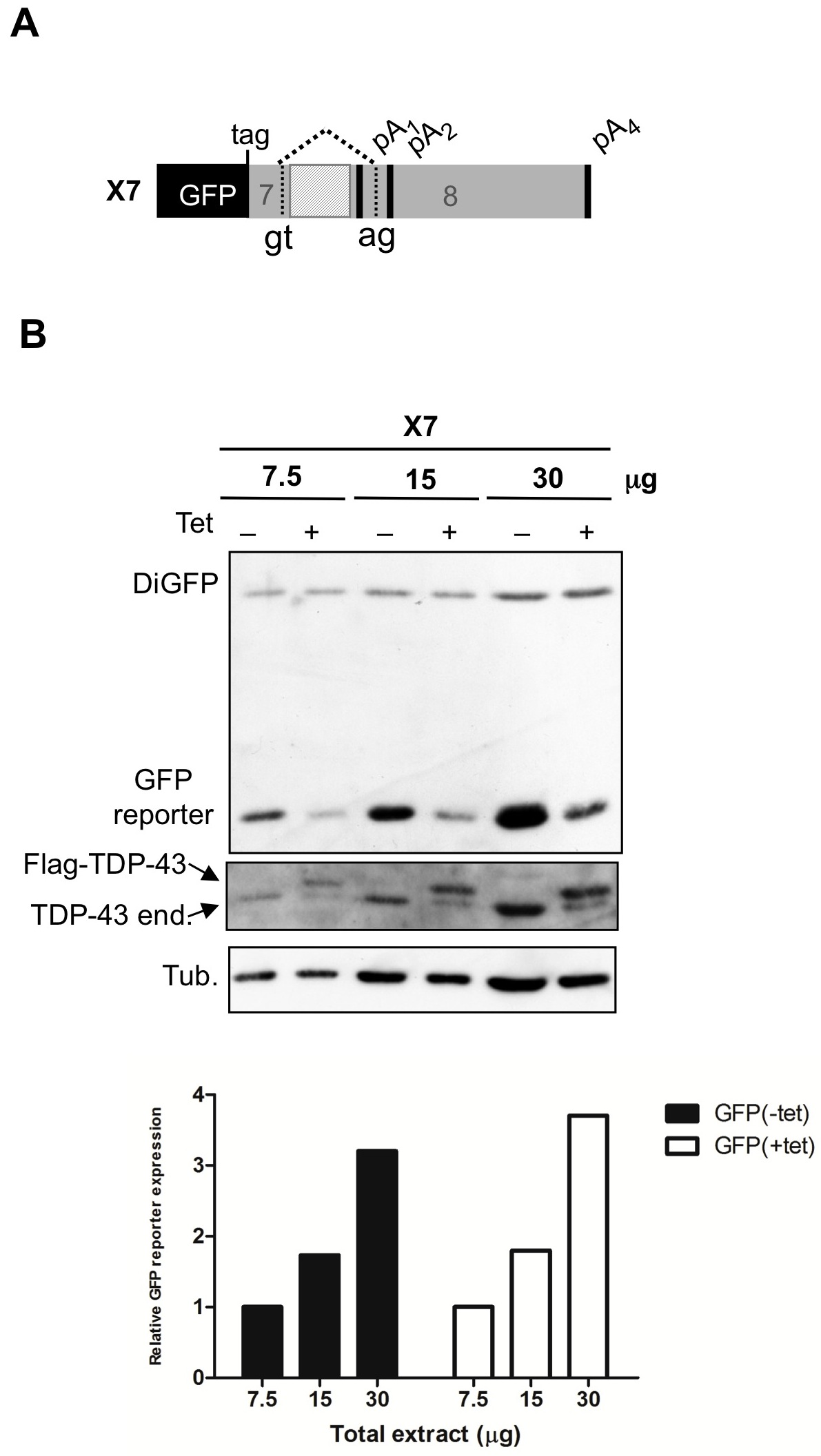


**Suppl.Fig.1 Calibration curve of the X7 construct showing a linear GFP antibody response in our experimental conditions.** With regards to GFP protein production, Suppl. Fig. 1 shows that in our experimental conditions (15 μg per lane) the response of the GFP protein from the X7 construct (Suppl.Fig.1A) under –Tet and +Tet conditions was in the linear response range. This construct was cotransfected with DiGFP to allow for internal normalization (Suppl.Fig.1B). Below this figure, a Western blot is also reported to show the correct overexpression of the transgene (Flag-TDP-43) and the consequent shut down of the endogenous TDP-43 (TDP-43 end.) in the +Tet conditions. Finally, the Western blot was also hybridized with an antibody against Tubulin to act as a further normalizing control. The bar chart at the bottom shows the quantification of three independent experiments to quantify GFP protein expression levels in –Tet and +Tet conditions normalized according to DiGFP expression. Mean values are reported on the bar chart and error bars indicate s.d. from at least three independent experiments.

**
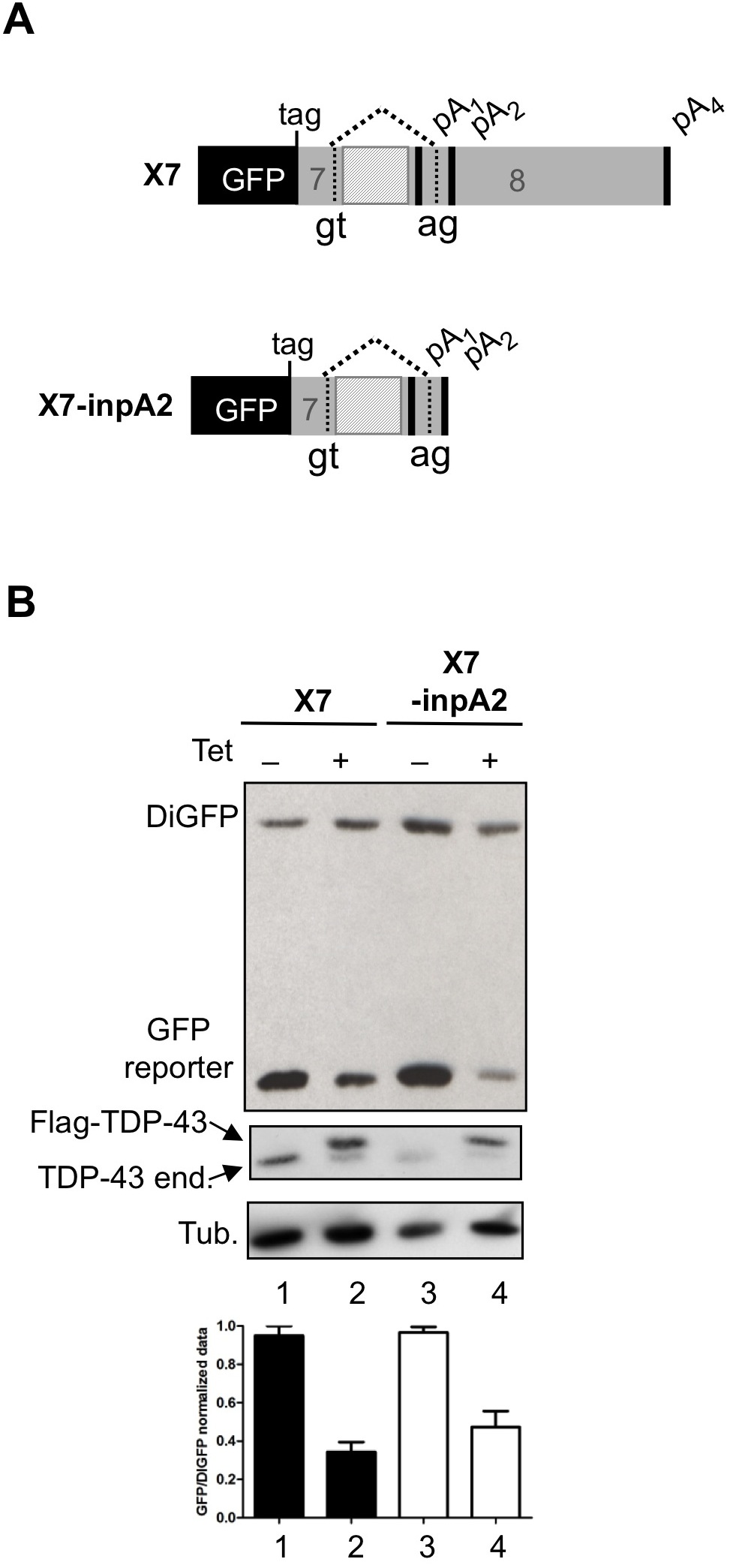
**

**Suppl.Fig.2 Comparing GFP protein expression in –Tet and +Tet conditions from the X7 and X7-inpA2 constructs.** Suppl.Fig.2A shows a schematic diagram of both the X7 and X7-inpA2 construct highlighting the fact that in the X7-inpA2 constructs the 3’UTR of TDP-43 is truncated just after the pA_2_ PAS site. With regards to GFP protein production, Suppl.Fig.2B shows GFP production from the X7-inpA2 construct compared to GFP production in the X7 construct, both –Tet and +Tet conditions. As in previous experiments, all these constructs were cotransfected with DiGFP to allow for internal normalization. Below this figure, a Western blot is also reported to show the correct overexpression of the transgene (Flag-TDP-43) and the consequent shut down of the endogenous TDP-43 (TDP-43 end.) in the +Tet conditions. Finally, the Western blot was also hybridized with an antibody against Tubulin to act as a further normalizing control. The bar chart at the bottom shows the quantification of three independent experiments to quantify GFP protein expression levels in –Tet and +Tet conditions normalized according to DiGFP expression. Mean values are reported on the bar chart and error bars indicate s.d. from at least three independent experiments.
